# Psychosocial Factors and Health: Community and Workplace Study

**DOI:** 10.2188/jea.15.65

**Published:** 2005-06-01

**Authors:** Akizumi Tsutsumi

**Affiliations:** 1 Okayama University Graduate School of Medicine, Dentistry and Pharmaceutical Sciences, Hygiene & Preventive Medicine.

**Keywords:** psychosocial factors, measurement, Social Support, Stress, intervention

## Abstract

The effects of psychosocial factors on health have drawn growing attention. An important prerequisite for epidemiologic studies is that instruments to measure psychosocial factors be reliable and valid based on psychometric properties. The introduction of occupational stress models has made breakthroughs in conceptualizing real-life complex phenomena in the workplace. This article describes some trials that explore the associations between psychosocial factors and health in the community and workplace. Scales for measuring social support and psychosocial job characteristics were developed, and their validation was pursued. Findings suggest that adverse social relationships and job characteristics measured by these instruments are associated with ill health. To strengthen the validities of the measurements and to provide strong causal evidence between psychosocial factors and health, more prospective studies and interventional approaches are needed.

Whether psychosocial factors affect health has drawn growing attention in current epidemiologic research. Through the use of psychometric techniques, improvements have been seen in the validity and reliability of the questionnaire-based instruments used to measure psychosocial factors.^1^ However, studies on the use of validated psychosocial questionnaires are scarce in Japanese populations. Some trials to fill knowledge gaps in the community and workplace are described in this article. The primary objective of the trials was to see the extent to which psychosocial factors are related to health risks. The validation of the measurement instruments was also pursued during the study.

## The Jichi Medical School (JMS) Cohort study

The JMS Cohort study is a prospective study that explores the risk factors of cardiovascular diseases among Japanese community residents.^2^ The baseline data were collected between 1992 and 1995. Ultimately, 12,490 Japanese from 12 communities located across Japan participated. The overall response rate was 65.4%.

The examined cardiovascular risk factors in the JMS Cohort study included traditional risk factors, such as behavioral factors (cigarette smoking, alcohol consumption, physical activity, and dietary patterns) and anthropometric / biological factors (body weight, height, blood pressure, blood glucose, serum lipids, and electrical cardiogram). The newly emerged risk factors, such as high-sensitivity C-reactive protein, coagulation factors, lipoprotein (a), and serum insulin, were also measured at baseline. Another unique objective of the JMS Cohort study was to investigate the health effects of psychosocial factors, which included social relationships of the individuals, type A behavior, and psychosocial job characteristics.

## Development of a social support scale

For a better understanding of social relationships and health, it is desirable to consider three aspects of social relationships: (1) their existence and number, (2) aspects of the network structure, and (3) functional content and quality of the relationships. Measures of the existence and number of social relationships or contacts, such as information on whether individuals are married, live alone, or belong to organizations, are relatively objective, reliable, and easy to obtain. Although more complicated, social network measurements or analyses in terms of size, density, and reciprocity are informative. However, current evidence postulates that the most sensible measure to tap the beneficial effects of social relationships is the function and quality of those relationships.^3^

We developed a 28-item instrument that measures the perceived availability of social support from a spouse, family, and friends for community residents involved in the JMS Cohort study.^4^^,^^5^ The internal consistencies were high, and both factorial and cross validities have been confirmed. In the cross-sectional analysis of 222 men and 417 women, perceived social support was shown to be generally associated with favorable health-related behavior.^6^ Among men, the support of friends was positively associated with the frequency of alcohol drinking; on the other hand, strong spousal support was related to reduced alcohol drinking, and enhanced family support was related to a reduction in smoking. Among women, family support was positively associated with the frequency of consuming Japanese food.

## Measurement of occupational stress

Stress is too vague a word to be informative in epidemiologic studies. For example, study participants are sometimes asked whether or not they experience occupational stress. The content may differ according to the individual. One person might perceive occupational stress as a result of time pressure, while another might suffer from a conflict in a personal relationship with a supervisor or colleague. Even if the exposure detected by the answer to such a question predicted a health outcome, it would be impossible to intervene because the precise nature of the stressor would be unknown. There have been improvements in the measurement of occupational stress, moving away from the generic idea of stress towards the combination of a few definite concepts based on theories. The introduction of stress models has made breakthroughs in occupational stress research and practices possible.

The job demand-control model is currently the most prevalent occupational stress model.^7^ The model posits that workers with a combination of high psychological job demands and low control over a job (i.e., job strain) have a risk of developing an illness.^8^ Although the combined effect of the demand and control components is of central interest in this model, its individual components may also predict a stress-related health outcome. To investigate the effects of adverse psychosocial job characteristics on cardiovascular diseases among Japanese workers, the JMS Cohort study adopted the MONICA version of the demand-control questionnaire, which was translated by Professor Uehata and his group.^9^ The internal consistency was acceptable for the job demands scale (*α* = 0.69) but slightly low for the job control scale (*α* = 0.65) in this cohort.

## Psychosocial job characteristics and cardiovascular diseases

Nearly 7,000 male and female workers in the JMS Cohort study were subjected to the analysis to investigate the extent to which psychosocial job characteristics by the demand-control model are related to cardiovascular risk factors. The working population was relatively older and healthier than the norm due to the recruitment method.^2^ Many of the individuals supposed to have long careers. In addition, the study population included large number of workers engaged in farming, forestry, and fisheries.

With a few exceptions, adverse job characteristics were related to unfavorable health behavior.^10^ High psychological demands were associated with heavy smoking, high prevalence of alcohol consumption, and high degrees of work-related physical activity. Low job control was associated with lower consumption of vegetables, a smaller number of cigarettes smoked, and a low level of work-related physical activity. Job strain, a combined measure obtained from the ratio of demands to control, was associated with lower vegetable consumption, low prevalence of smoking, and high prevalence of current alcohol consumption.

In men, a multiple logistic regression model revealed that job strain was significantly related to hypertension (odds ratio, 1.2; 95% confidence interval: 1.1-1.3), after adjusting for age, employment status (white-collar versus blue-collar), marital status, family history of hypertension, cigarette smoking, alcohol consumption, physical activity, and body mass index.^11^ This study also replicated the findings often cited in studies in Western countries, i.e., that the effects of the adverse job characteristics were stronger among workers in the lower socioeconomic strata.^12^ Significantly greater risks were observed among employees in subordinate positions, blue-collar jobs and the less-educated, than among those in the respective counterparts.

Higher psychological job demands were associated with a higher total cholesterol level, with an adjusted difference from the top to bottom tertile of 3.3 mg/dL (F = 3.03; p = 0.048). Higher job demands were also significantly associated with the total/HDL cholesterol ratio (F = 3.94; p = 0.020).^13^ However, the magnitude of the observed effect, up to a 3% average difference of the total cholesterol levels, may not be clinically meaningful.

Studies from Western industrialized societies have demonstrated that the job demand-control model predicts cardiovascular diseases, in particular, ischemic heart disease. In Japan, stroke is an important heavy burden on the public. Based on the ongoing data from the JMS Cohort study, we examined the extent to which psychosocial job characteristics are related to the risk of stroke (Unpublished data). The participants were followed 8 years on average until March 2002 using systematic surveillance. Incidence cases were identified according to criteria established by the Japan Ministry of Health and Welfare. Cox proportional hazards regression analysis was used for multivariate analyses.

Among the participants who had been free from stroke at baseline, 97 cases were identified during the follow-up. Workers exposed to job strain (concurrent high job demands and low job control) had the highest risk of stroke. Compared with workers in low strain jobs (low job demands and high job control), the age- and sex-adjusted relative risk of stroke was a hazard ratio = 1.4 (95% confidence interval: 0.7-2.7); the point estimate is compatible with recent Western reports on the relative risk of adverse job strain on ischemic heart disease or cardiovascular death.^14^ The adjustment for socioeconomic status, behavioral, and biological risk factors slightly reduced the risk. These statistically insignificant findings may be attributable to the small number of outcome events, possibly due to survivor bias or insufficient follow-up periods, and the relatively low scale reliability.

The findings suggest that psychosocial job characteristics defined by the job demand-control model predict cardiovascular diseases among Japanese workers. However, the inconclusive findings to date warrant further investigation.

## A new occupational stress model

Another more recently developed occupational stress model is the effort-reward imbalance (ERI) model.^15^ This model postulates that the source of stress at the workplace results from an imbalance between an individual’s recognition of his extrinsic effort (e.g., high workload) and the extrinsic reward (e.g., money, esteem, and occupational status control), with a focus on a negative trade-off between experienced costs and gains at work. In the current labor market, socioeconomic factors beyond an individual’s ability to change them have the potential to produce a significant amount of stress. That is, economic recession and globalization have lead to organizational restructuring, which includes downsizing. Job insecurity has been identified as a serious health risk. Thus, the ERI model is expected to capture the current occupational stress most sensitively. Although this model has been studied much less than the job demand-control model, evidence showing that ERI predicts cardiovascular diseases has been accumulating.^7^

The Japanese version of the ERI questionnaire was developed through a back-translation process.^16^ The reliability was acceptable for various Japanese working populations, and the validity has been confirmed in several investigations at workplaces.

The responsiveness of the stress measures was demonstrated in a real case of company restructuring.^17^ For the employees who had been affected by restructuring of their company due to economic hardship, two consecutive questionnaire surveys were conducted over a specific period. A total of 544 full-time employees responded to both surveys. Changes in the summary measures from the ERI model component were evaluated. The summary measures showed significant psychological deterioration in the total study population. The deterioration was prevalent in those employees who had presumably experienced the effects of stressful organizational changes related to the restructuring; potentially stronger effects of multiple organizational changes on employees were indicated.

The complementary roles of the effort-reward imbalance and the job demand-control models have been clarified; the two models identify different aspects of occupational stress and that the health effects are independent of each other.^18^ A cross-sectional analysis was conducted to examine these associations in 190 male and female workers of a small Japanese plant with economic hardship. Workers were engaged in two types of jobs – direct assembly line and indirect support tasks –; the latter was threatened by job loss because of downsizing. Independent variables were measured by the Japanese versions of the demand-control and ERI questionnaires. The Center for Epidemiologic Studies Depression Scale was used to assess depressive symptomatology. Workers with indirect supportive tasks (target for downsizing) were more likely to have depressive symptoms than assembly line workers. Job strain, a combination of high demand and low control at work, was more frequent among the assembly line workers, while the combination of high effort and low reward was more frequent among the workers with indirect supportive tasks. After adjusting for work environmental factors, low control and ERI were independently related to depressive symptoms (odds ratios 4.7 and 4.1, respectively).

Other than the above-mentioned studies, the criterion validity was tested in terms of the associations with psychological and physiological health outcome; the ERI stress indices were associated with several psycho-physiological and behavioral health outcomes. The discriminant validity was discussed in terms of the stress prevalence in various socioeconomic strata.^19^

## Intervention

Although not based on specific stress models, our trial provided a suggestive evidence that providing supervisors with appropriate information with regard to mental health at the workplace, including occupational stress models, has a positive effect on employee psychological well-being.^20^

A single-session supervisory education program was developed in conjunction with the Japanese national guidelines for the promotion of employee mental health. A total of 267 voluntary supervisors in a prefectural office were presented with comprehensive information on the role they are to fulfill to promote mental health in the workplace. A total of 864 office employees were evaluated to determine whether education had had an effect on their psychological distress. Three months after the education, the levels of psychological distress improved among employees in the departments in which more than one-third of the supervisors attended an educational session; these findings were compared to those from departments with lower attendance rates of the supervisors. The positive effect of education was supported by statistically significant interaction between time and the category of the department; adjustments were made for the confounders.

## Conclusions

Evidence from communities and workplaces suggests that psychosocial factors affect health among Japanese populations. Accurate measurements of psychosocial factors are a crucial prerequisite to obtain rigorous evidence. Prospective and interventional studies are necessary for more definitive conclusions. Well-defined occupational stress models serve as useful tools for implementing theory-based stress-reduction approaches by dealing with real-life complex phenomena in the workplace.^21^^,^^22^ Successful intervention will further strengthen the validities of the measurements and models as well as provide strong causal evidence between psychosocial factors and health.
